# Predicting Amyloid Burden to Accelerate Recruitment of Secondary Prevention Clinical Trials

**DOI:** 10.14283/jpad.2020.44

**Published:** 2020

**Authors:** O. Langford, R. Raman, R.A. Sperling, J. Cummings, C.-K. Sun, G. Jimenez-Maggiora, P.S. Aisen, M.C. Donohue, TRC-PAD Investigators

**Affiliations:** 1. Alzheimer’s Therapeutic Research Institute, University of Southern California, San Diego, CA, USA; 2. Center for Alzheimer Research and Treatment, Brigham and Women’s Hospital, Harvard Medical School, Boston, MA, USA; 3. Chambers-Grundy Center for Transformative Neuroscience, Department of Brain Health, School of Integrated Health Sciences, University of Nevada, Las Vegas; Cleveland Clinic Lou Ruvo Center for Brain Health, Las Vegas, NV, USA

**Keywords:** Trial-ready cohort, Alzheimer’s disease, machine learning

## Abstract

**BACKGROUND:**

Screening to identify individuals with elevated brain amyloid (Aβ+) for clinical trials in Preclinical Alzheimer’s Disease (PAD), such as the Anti-Amyloid Treatment in Asymptomatic Alzheimer’s disease (A4) trial, is slow and costly. The Trial-Ready Cohort in Preclinical/Prodromal Alzheimer’s Disease (TRC-PAD) aims to accelerate and reduce costs of AD trial recruitment by maintaining a web-based registry of potential trial participants, and using predictive algorithms to assess their likelihood of suitability for PAD trials.

**OBJECTIVES:**

Here we describe how algorithms used to predict amyloid burden within TRC-PAD project were derived using screening data from the A4 trial.

**DESIGN:**

We apply machine learning techniques to predict amyloid positivity. Demographic variables, APOE genotype, and measures of cognition and function are considered as predictors. Model data were derived from the A4 trial.

**SETTING:**

TRC-PAD data are collected from web-based and in-person assessments and are used to predict the risk of elevated amyloid and assess eligibility for AD trials.

**PARTICIPANTS:**

Pre-randomization, cross-sectional data from the ongoing A4 trial are used to develop statistical models.

**MEASUREMENTS:**

Models use a range of cognitive tests and subjective memory assessments, along with demographic variables. Amyloid positivity in A4 was confirmed using positron emission tomography (PET).

**RESULTS:**

The A4 trial screened N=4,486 participants, of which N=1323 (29%) were classified as Aβ+ (SUVR ≥ 1.15). The Area under the Receiver Operating Characteristic curves for these models ranged from 0.60 (95% CI 0.56 to 0.64) for a web-based battery without APOE to 0.74 (95% CI 0.70 to 0.78) for an in-person battery. The number needed to screen to identify an Aβ+ individual is reduced from 3.39 in A4 to 2.62 in the remote setting without APOE, and 1.61 in the remote setting with APOE.

**CONCLUSIONS:**

Predictive algorithms in a web-based registry can improve the efficiency of screening in future secondary prevention trials. APOE status contributes most to predictive accuracy with cross-sectional data. Blood-based assays of amyloid will likely improve the prediction of amyloid PET positivity.

## Background

Screening cognitively normal older individuals for the presence of elevated cerebral amyloid-beta protein (“Aβ+”) and inclusion in secondary prevention trials for Alzheimer’s disease (AD) is invasive, expensive and slow. The current gold standards to measure Amyloid-β in the brain require either positron emission tomography (PET) or cerebrospinal fluid (CSF) assay. For example, the Anti-Amyloid Treatment in Asymptomatic Alzheimer’s disease (A4) trial conducted amyloid PET on 4,486 individuals in order to identify 1,323 Aβ+ individuals for an amyloid PET screen fail rate of 71% (1). The Number Needed to Screen (NNS) to identify each Aβ+ individual was 3.39 individuals.

Trial-Ready Cohort in Preclinical/Prodromal Alzheimer’s Disease (TRC-PAD) is a research program that was initiated to find solutions to these challenges in trial recruitment and site management, as described in Aisen, et al. Submitted (2). There are three elements that make up the TRC-PAD platform; Alzheimer’s Prevention Trial (APT) webstudy (aptwebstudy.org), Site Referral System (SRS) and the Trial Ready Cohort (TRC). The APT webstudy invites participants to enroll into the study. At the time of enrollment, participants are asked for demographic, medical and lifestyle information. They are asked to complete longitudinal web-based cognitive testing and symptom questionnaires. With these data, we aim to estimate the likelihood that an individual is Aβ+ before they are invited to participate in a secondary prevention trial. The SRS helps facilitate the participants deemed to be most likely Aβ+ from APT to go for in-clinic assessments where they proceed with the TRC screening. During the TRC screening phase participants are administered additional testing, including Preclinical Alzheimer’s Cognitive Composite (PACC) (3) and genotyping, before assessing their eligibility for an amyloid test.

In this paper, we describe how the prediction models and algorithms used in TRC-PAD were derived from A4 screening data. We anticipate blood-based biomarkers will greatly improve predictions of amyloid positivity, and this is a focus of future work and an aim of TRC-PAD. Predictors in the current analysis are limited to demographics, cognitive and functional assessments, and APOE genotype.

## Methods

### Population and Study Design

The study design and screening data for A4 have been previously described (7, 8) and Institutional Review Boards have approved both A4 and TRC-PAD studies. The A4 screening dataset contains N=4,486 participants, of which 1323 (29%) were classified as Aβ+. Amyloid PET imaging was conducted with florbetapir F18 and summarized by mean cortical standardized uptake value ratio (SUVR) relative to the whole cerebellum. Participants were considered eligible to continue screening for A4 based on an algorithm combining both quantitative SUVR (≥1.15) and qualitative visual read performed at a central laboratory. A SUVR between 1.10 and 1.15 was considered to be elevated amyloid only if the visual read was considered positive by a two-reader consensus determination. Participants who were considered Aβ+ were slightly older; with mean/standard deviation (SD) age of 72.10/4.89 in the Aβ+ group and 70.95/4.53 in the Aβ- group. However, there were no observed differences in sex and education. Aβ+ participants were more likely to have a family history of dementia and at least one APOEε4 allele. In addition, Aβ+ participants performed worse on the screening Preclinical Alzheimer Cognitive Composite (PACC) results and had higher scores on the Cognitive Function Index.

### Variables

Table 1 describes the collections of predictors that we considered to train different predictive algorithms. All screening data for the A4 Study were collected during supervised clinic visits. However some components of the A4 screening battery are being captured remotely in the APT webstudy, including demographic, Cogstate brief battery (9), family history (sibling or parent with Alzheimer’s), and Cognitive Function Instrument (10) (CFI) variables indicated in Table 1. We consider predictive algorithms using these “remote” variables only, as well as a more thorough battery that would require a supervised clinic visit with an administration of the PACC3. In all, we considered 6 models: (1) remote battery without APOE, (2) remote battery with APOE, (3) in clinic battery without APOE, (4) in clinic battery with APOE, (5) in clinic battery with individual PACC component scores without APOE, and (6) in clinic battery with individual PACC component scores with APOE. The PACC component scores include the Mini-Mental State Exam (MMSE) (11), Wechsler Memory Scale-Revised Logical Memory, Digit Symbol Substitution (DSST), and Free and Cued Selective Reminding Test (FCSRT) (12).

### Statistical Analysis

Extreme Gradient Boosting (XGBoost) (4) is a decision tree-based machine learning technique (6). A single decisions tree, or regression tree, is easy to interpret but provides relatively poor prediction. Aggregating a large number of trees can improve prediction accuracy. Boosting is a technique in which models are trained in sequence, with each new model making cumulative improvements. At each iteration the data are re-weighted such that misclassified data points receive larger weights. XGBoost is a scalable tree boosting algorithm, that is optimized and designed to be highly efficient, flexible, and portable.

XGBoost supports monotone constraints and customized objective functions. We applied monotone constraints to predictors such as age, number of APOEε4 alleles (0, 1 or 2), and assessment scores that we expect to have a generally monotonic relationship with amyloid PET SUVR (Supplemental Figure 1). The default XGBoost objective function is mean squared loss, meaning decision trees are selected to minimize the residual sum of squares. Because XGBoost does not provide confidence intervals with mean squared loss, we applied the Quantile Regression loss function to estimate the 50%, 2.5%, and 97.5% quantiles of the predictions. XGBoost model has a number of hyper-parameters that are used to assist in the issue known as the bias-variance trade-off (13). Hyper-parameters are fixed before the model is fitted and are not learned from data. We used 10-fold Cross-Validation (CV) to assess the out-of-sample bias and variance for given hyper-parameter values, and Bayesian Optimization (14) to optimize the hyper-parameter selection. We use SHapley Additive exPlanation (SHAP) (15) values to summarize the importance of each predictor to the overall predictive accuracy of each model. More details about the model fitting procedures are provided in the supplemental material (Supplemental Table 1). Our main interest lies in the predictive accuracy of the models. In order to assess this, we split the data randomly into 80% training and 20% test. After fitting the models with the training data, we assess their predictive accuracy with the independent test data. Analyses were conducted with R version 3.6.2 (r-project.org) with packages xgboost (4) version 0.90.0.2 and mlrMBO (16) version 1.1.2.

## Results

**Figure 1.**
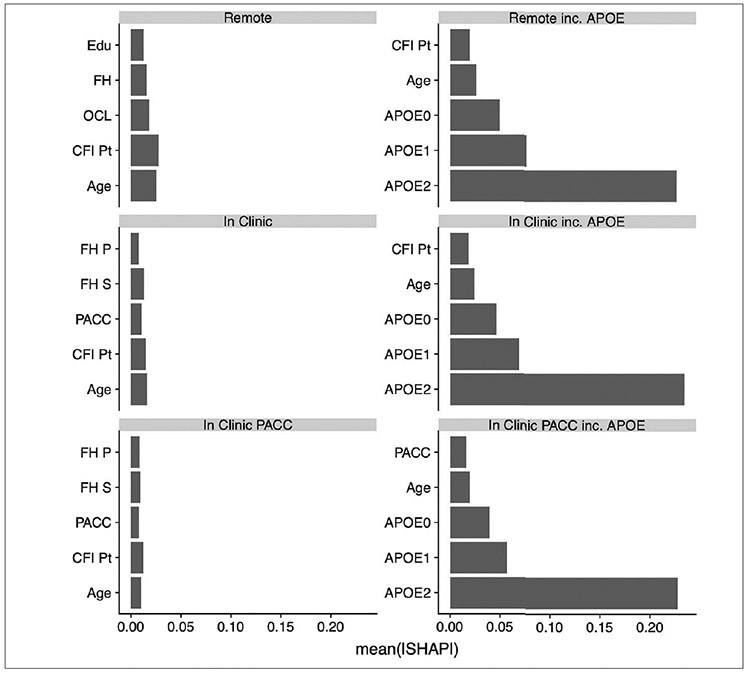
Contribution of 5 best predictors in each model Using the model training data we see the contribution to prediction accuracy expressed in terms of the mean absolute SHAP value (mean ∣SHAP∣). Abbreviations: SHAP, SHapley Additive explanation; OCL, One Card Learning; OBR, One Back Reaction; DER, Detection Reaction; IDR, Identification Reaction; FH, Family History; FH P, FH Parent; FH S, FH Sibling; CFI, Cognitive Function Index; CFI Pt, CFI Participant; CFI SP, CFI Study Partner; ADL, Activities of Daily Living; ADL Pt, ADL Participant; ADL SP, ADL Study Partner; PACC, Preclinical Alzheimer Cognitive Composite

**Figure 2.**
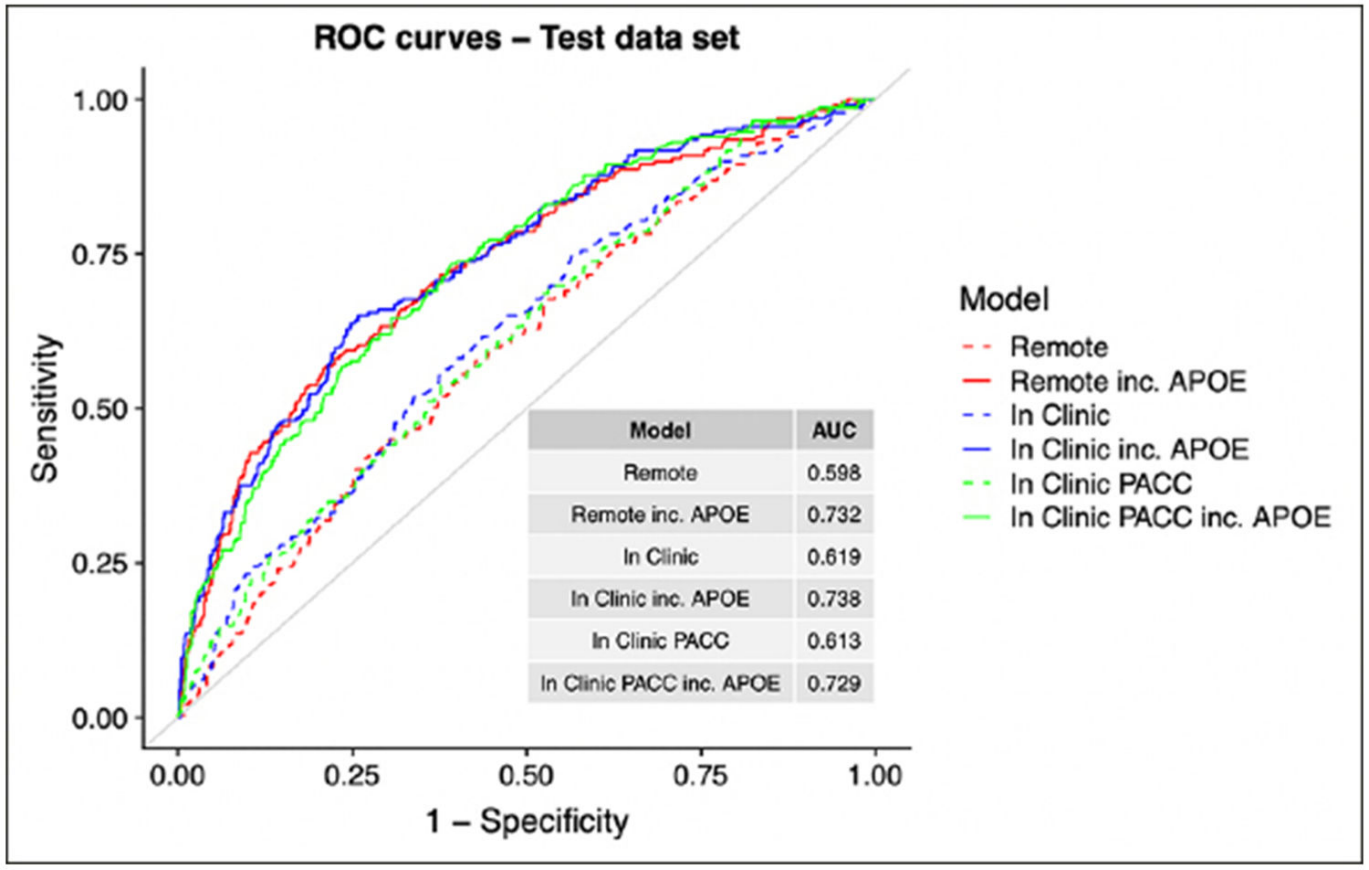
ROC curves and AUCs ROCs and AUCs for each model are determined using the independent test set and Aβ+ set to SUVR ≥ 1.15. The colors represent the setting type; Remote (red), In-Clinic (blue) and PACC components (blue). Abbreviations: ROC, Receiver Operating Characteristic; AUC, Area Under the Curve; PACC, Preclinical Alzheimer Cognitive Composite

Figure 1 shows the relative contributions, in terms of SHAP values, for each predictor to the predictive accuracy of each model. As expected, when available, APOE genotype is the most important predictor for these cross-sectional models. We see that age, CFI, education, and family history also enter the top 5 most valuable predictors in some models. Figure 2, the Receiver Operating Characteristic (ROC) curves and Area under the Curve (AUC) for the 6 models, also demonstrates the relative value of APOE. The dashed lines are models fitted without the APOEε4 variable and the solid lines are for models that include APOEε4. The ROC curves were generated using a cut point SUVR value of 1.15 for a binary separation between amyloid positive and negative. In general, we see AUCs in the range 0.60 (without APOE) to 0.73 (with APOE).

**Figure 3.**
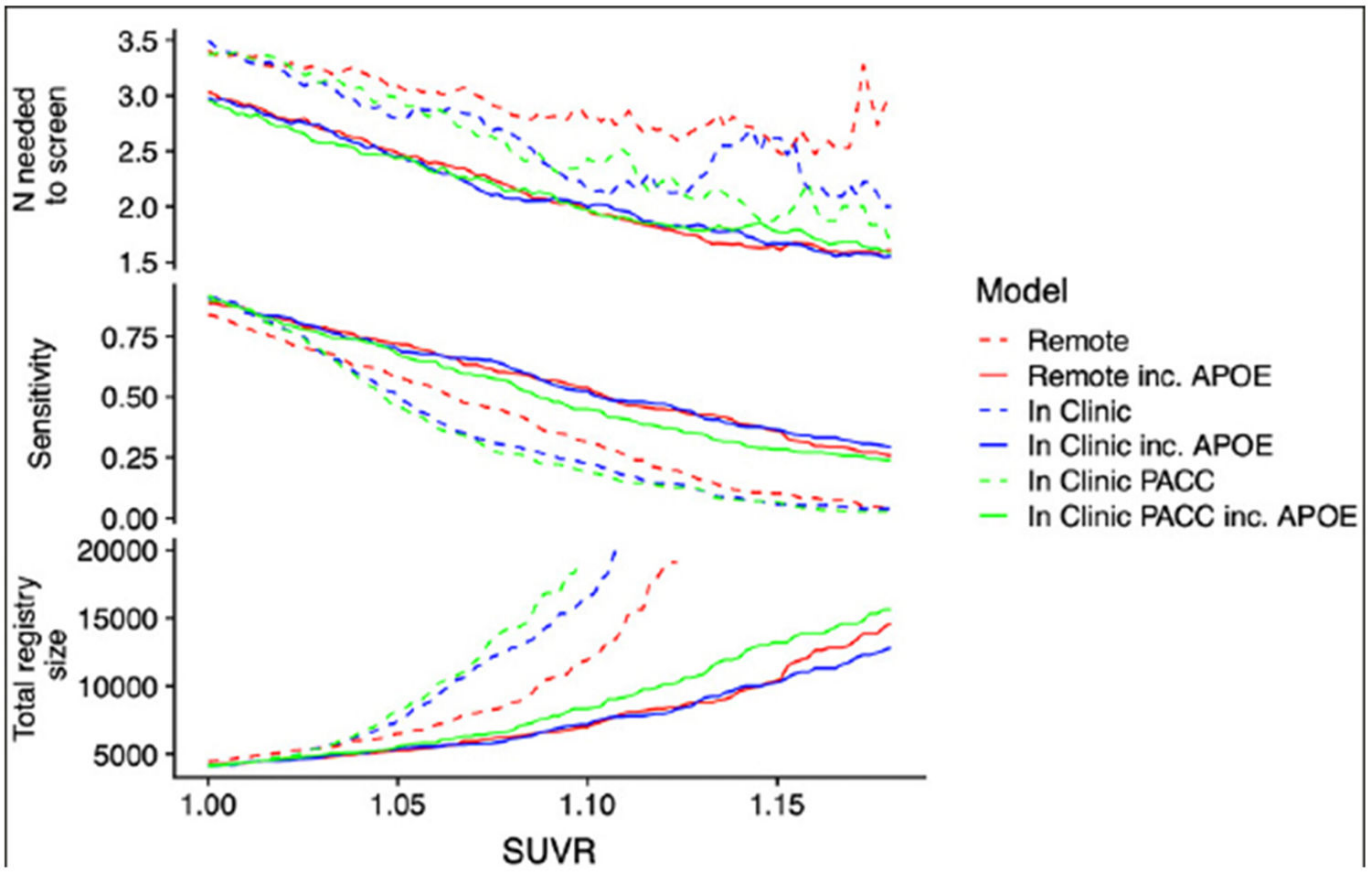
Number needed to screen and required registry size The top panel shows the number needed to screen (which is equivalent to 1/PPV) with amyloid PET to identify one Aβ+ individual by applying the given SUVR threshold to the values predicted from each model. The middle panel shows sensitivity. The models not containing APOEε4 all have lower sensitivity. The bottom panel shows the size of the screening pool (e.g. web-based registry) that would be required to recruit 1,000 Aβ+ individuals by applying the given SUVR threshold to values predicted from each model. Abbreviations: PPV, Positive Predictive Value; SUVR, Standardized Uptake Value Ratio; PACC, Preclinical Alzheimer Cognitive Composite; PET, positron emission tomography

Figure 3 expresses prediction accuracy in terms of screening for a clinical trial. The top panel shows 1/Positive Predictive Value (PPV), which is equivalent to the number needed to screen (with amyloid PET) to identify one eligible participant. In this figure, movement along the horizontal axis represents varying the threshold applied to SUVRs predicted from each model. The bottom panel provides the required number of potential participants (e.g. webstudy participants) in order to identify 1,000 Aβ+ participants.

Table 2 reports operating characteristics from several screening algorithm scenarios. The top half provides operating characteristics when a threshold is selected to provide 50% prediction prevalence (i.e. select half the participant pool to receive amyloid PET scans). With 50% prediction prevalence, the NNS is about 2.5 participants with APOE and 3.0 participants without APOE. When the threshold for predicted amyloid PET is increased to 1.15, the NNS is reduced to about 1.7 participants with APOE and 2.5 participants without APOE. However, this results in much lower sensitivity, and as we can see from Figure 3, a threshold of 1.15 would be practical only with participant registries of 10,000-13,000 to identify 1,000 Aβ+ participants.

## Discussion

This work, in the context of the TRC-PAD platform, can facilitate the development of participant selection algorithms. TRC-PAD has two main selection points; the first is from the APT webstudy to in-clinic assessment (stage 1) and the second is from in-clinic to amyloid testing (stage 2). In stage 1, consented webstudy participants are referred to their nearest TRC-PAD site, identified via the use of self-reported zip codes. They are then ranked based on their SUVR prediction. In addition to this predicted SUVR, the selection process considers demographics to achieve diversity and if the participant has known prior amyloid testing and results. During the first in-clinic visit of the referred participants in stage 1, additional cognitive testing, in the form of the PACC, and APOE genotyping is performed. With this additional information, the SUVR predictions are updated and presented for central authorization of amyloid testing.

This work has shown that by collecting relatively simple demographics, cognitive and functional assessments remotely, via the webstudy, we will be able to reduce screen fail rates and improve enrollment. Even small improvements in NNS can have a large impact on the expense of screening for Preclinical AD clinical trials. For example, assuming a conservative estimate of 3,500 US Dollars (USD) per scan, the A4 study spent a total of about 4,486x3,500 (USD) = 15,701,000 (USD) for screening amyloid PET scans alone to identify 1,323 Aβ+ individuals (NNS=3.39). Reducing the NNS from 3.39 to 2.62, which seems plausible with the simplest remote battery, would have reduced this cost by 3,569,090 (USD) to 1,323x2.62x3,500 (USD) = 12,131,910 (USD). In addition to the remote data setting, this work included the value of APOE genotyping and collection of PACC during an in-clinic screening. Adding APOE genotype might reduce NNS to below 2.00, for a total PET screening cost of 1,323x2.00x3,500 (USD) = 9,261,000 (USD). The financial impact would be less with a cerebrospinal fluid (CSF)-based, or blood-based, amyloid screen, but the impact on subject and site burden would remain significant. From a statistical aspect, we have demonstrated the use of Machine Learning Techniques to both optimize, via Bayesian Optimization, and produce predictive models using XGBoost. We have illustrated how to make inferences from a modelling approach that is primarily used for prediction via the SHAP metric.

One limitation of these pre-screening algorithms is that the cohort characteristics will be impacted. For example, we would expect the algorithms to produce an older cohort with an even greater proportion of APOEε4 carriers than a cohort selected without a pre-screen. This could be mitigated by stratifying the screening process to ensure an adequate sample of younger, APOEε4 noncarriers; but with adverse effects on the NNS. Another consideration is the inability for these models to extrapolate beyond the data in the continuous variables such as age. A second potential limitation is in the bias of the training data. As we start using these models in TRC-PAD and collect additional data, we will assess whether the models are biased against any additional covariates collected.

Future work will focus on utilizing longitudinal cognitive and functional change and/or the use of blood-based biomarkers to improve the performance of these predictive models and algorithms. We anticipate, based on analyses of the Alzheimer Disease Neuroimaging Initiative (ADNI) (5), that longitudinal change may be a valuable predictor of amyloid status. In addition, we will incorporate plasma amyloid peptide ratios (currently in validation testing) into the final stage of prediction and expect a large improvement in prediction.

## Supplementary Material

1652725_SuppMaterial

## Figures and Tables

**Table 1.** Predictors Considered

Abbreviation	Variable	Description	Remote	In Clinic
Age	Age	Number of years	√	√
Edu	Education	Number of years	√	√
Sex	Sex	Male or Female	√	√
OCL	Cogstate One Card Learning	Accuracy	√	
OBR	Cogstate One Back Reaction	Reaction time	√	
DER	Cogstate Detection Reaction	Reaction time	√	
IDR	Cogstate Identification Reaction	Reaction time	√	
FH	Family History	Family history a parent or sibling with AD	√	
FH P	Family History - Parent	Family history a parent with AD		√
FH S	Family History - Sibling	Family history a sibling with AD		√
CFI Pt	Cognitive Function Instrument - Participant		√	√
CFI SP	Cognitive Function Instrument - Study Partner			√
ADL Pt	Activities of Daily Living - Participant			√
ADL SP	Activities of Daily Living - Study Partner			√
PACC	Preclinical Alzheimer Cognitive Composite			√
APOE4	APOEε4		(*X*/√)	(*X*/√)

We considered predictive algorithms which could be applied to data captured either remotely via a web-based registry, or in the clinic (though all data in A4 was collected in clinic), as indicated in the table. In all we considered 6 models: (1) remote battery with APOE, (2) remote battery without APOE, (3) in clinic battery with APOE, (4) in clinic battery without APOE, (5) in clinic battery with individual PACC component scores and APOE, and (6) in clinic battery with individual PACC component scores without APOE.

**Table 2.** Operating characteristics of screening algorithms using the test data with Aβ+ set to SUVR ≥ 1.15

Model	SUVR Threshold	Accuracy	Sensitivity	Specificity	NPV	PPV	NNS
Remote	1.05	54.88%	60.70%	52.77%	78.72%	31.81%	3.14
Remote with APOE	1.04	61.86%	74.24%	57.37%	85.99%	38.72%	2.58
In Clinic	1.04	57.21%	62.45%	55.31%	80.23%	33.65%	2.97
In Clinic with APOE	1.04	62.33%	73.80%	58.16%	85.95%	39.03%	2.56
In Clinic PACC	1.04	56.63%	58.95%	55.78%	78.92%	32.61%	3.07
In Clinic PACC with APOE	1.04	62.67%	73.80%	58.64%	86.05%	39.30%	2.54
Remote	1.15	71.98%	10.04%	94.45%	74.31%	39.66%	2.52
Remote with APOE	1.15	77.09%	35.81%	92.08%	79.81%	62.12%	1.61
In Clinic	1.15	72.44%	5.68%	96.67%	73.85%	38.24%	2.62
In Clinic with APOE	1.15	76.51%	36.24%	91.13%	79.75%	59.71%	1.67
In Clinic PACC	1.15	73.60%	6.55%	97.94%	74.28%	53.57%	1.87
In Clinic PACC with APOE	1.15	75.00%	28.38%	91.92%	77.96%	56.03%	1.78

The top half of the table provides operating characteristics when a threshold is applied to predicted amyloid PET SUVR that results in a 50% prediction prevalence (half of the screening pool is predicted positive and tested with a PET scan). The first column indicates the threshold required to attain 50% prediction prevalence. The bottom half of the table applies a threshold of 1.15, which reduces Number Need to Screen (NNS), but also greatly reduces sensitivity. The NNS is the inverse of the Positive Predictive Value (PPV). The PPV indicates the percentage of participants that are truly positive when the model indicates them as positive. Likewise, the Negative Predictive Value (NPV), this gives the probability that a participant is truly amyloid negative when the model indicates them as negative.

**Table 3.** Demographic characteristics of amyloid positive selections from the test data with Aβ+ set to SUVR ≥ 1.15

		APOE4			Family History		Sex	
Model	SUVR Threshold	0	1	2	No	Yes	Female	Male
Remote	1.05	298 (68.19%)	123 (28.15%)	16 (3.66%)	140 (32.04%)	297 (67.96%)	248 (56.75%)	189 (43.25%)
Remote with APOE	1.04	169 (38.50%)	240 (54.67%)	30 (6.83%)	117 (26.65%)	322 (73.35%)	248 (56.49%)	191 (43.51%)
In Clinic	1.04	283 (66.59%)	128 (30.12%)	14 (3.29%)	112 (26.35%)	313 (73.65%)	248 (58.35%)	177 (41.65%)
In Clinic with APOE	1.04	175 (40.42%)	228 (52.66%)	30 (6.93%)	120 (27.71%)	313 (72.29%)	240 (55.43%)	193 (44.57%)
In Clinic PACC	1.04	281 (67.87%)	119 (28.74%)	14 (3.38%)	106 (25.60%)	308 (74.40%)	246 (59.42%)	168 (40.58%)
In Clinic PACC with APOE	1.04	177 (41.16%)	223 (51.86%)	30 (6.98%)	100 (23.26%)	330 (76.74%)	256 (59.53%)	174 (40.47%)
Remote	1.15	39 (67.24%)	17 (29.31%)	2 (3.45%)	6 (10.34%)	52 (89.66%)	27 (46.55%)	31 (53.45%)
Remote with APOE	1.15	6 (4.55%)	96 (72.73%)	30 (22.73%)	30 (22.73%)	102 (77.27%)	67 (50.76%)	65 (49.24%)
In Clinic	1.15	26 (76.47%)	8 (23.53%)	0 (0.00%)	7 (20.59%)	27 (79.41%)	15 (44.12%)	19 (55.88%)
In Clinic with APOE	1.15	22 (15.83%)	88 (63.31%)	29 (20.86%)	34 (24.46%)	105 (75.54%)	67 (48.2%)	72 (51.80%)
In Clinic PACC	1.15	16 (57.14%)	12 (42.86%)	0 (0.00%)	4 (14.29%)	24 (85.71%)	16 (57.14%)	12 (42.86%)
In Clinic PACC with APOE	1.15	18 (15.52%)	68 (58.62%)	30 (25.86%)	27 (23.28%)	89 (76.72%)	55 (47.41%)	61 (52.59%)

The top half of the table provides demographic characteristics when a threshold is applied to predicted amyloid PET SUVR that results in a 50% prediction prevalence (half of the screening pool is predicted positive and tested with a PET scan). The first column indicates the threshold required to attain 50% prediction prevalence. The bottom half of the table applies a threshold of 1.15. We can see in all the scenarios where APOE is included in the model, at least 29 of the 30 participants with APOE4 2 allele (in the test data) have been selected.

## References

[1] Sperling RA, Donohue MC, Raman R, Association of Factors With Elevated Amyloid Burden in Clinically Normal Older Individuals. JAMA Neurol. 2020. 10.1001/jamaneurol.2020.0387 PMC7136861 32250387

[2] Aisen PS, Sperling RA, J Cummings J, The Trial-Ready Cohort for Preclinical/prodromal Alzheimer’s Disease (TRC-PAD) Project: An Overview. J Prev Alz Dis 2020;4 (7):208–212 10.14283/jpad.2020.45 PMC7735207 32920621

[3] Donohue MC, Sperling RA, Salmon DP, The Preclinical Alzheimer Cognitive Composite: measuring amyloid-related decline. JAMA neurology. 2014;71 (8):961–970. 24886908 10.1001/jamaneurol.2014.803 PMC4439182

[4] Chen T, He T, Benesty M, xgboost: Extreme Gradient Boosting. 2018.

[5] Insel PS, Palmqvist S, Mackin RS, Assessing risk for preclinical β-amyloid pathology with APOE, cognitive, and demographic information. Alzheimer’s & Dementia: Diagnosis, Assessment & Disease Monitoring. 2016;4:76–84. 10.1016/j.dadm.2016.07.002 PMC5045949 27722193

[6] Breiman L Random forests. Machine learning. 2001;45 (1):5–32.

[7] Sperling RA, Rentz DM, Johnson KA, The A4 study: stopping AD before symptoms begin? Science translational medicine. 2014;6 (228):228fs213. 10.1126/scitranslmed.3007941 PMC4049292 24648338

[8] Sperling RA, Donohue MC, Raman R, Factors associated with elevated amyloid burden in cognitively unimpaired older individuals: Screening Amyloid PET results from the A4 Study. Submitted.

[9] Maruff P, Thomas E, Cysique L, Validity of the CogState brief battery: relationship to standardized tests and sensitivity to cognitive impairment in mild traumatic brain injury, schizophrenia, and AIDS dementia complex. Archives of Clinical Neuropsychology. 2009;24 (2):165–178. 19395350 10.1093/arclin/acp010

[10] Walsh SP, Raman R, Jones KB, Aisen PS. ADCS Prevention Instrument Project: the Mail-In Cognitive Function Screening Instrument (MCFSI). Alzheimer Dis Assoc Disord. 2006;20 (4 Suppl 3):S170–178. 17135810 10.1097/01.wad.0000213879.55547.57

[11] Folstein MF, Folstein SE, McHugh PR. “Mini-mental state”: a practical method for grading the cognitive state of patients for the clinician. Journal of psychiatric research. 1975;12 (3):189–198. 1202204 10.1016/0022-3956(75)90026-6

[12] Buschke H Cued recall in amnesia. J Clin Neuropsychol. 1984;6 (4):433–440. 6501581 10.1080/01688638408401233

[13] James G, Witten D, Hastie T, Tibshirani R. An introduction to statistical learning. Vol 112: Springer; 2013.

[14] Snoek J, Larochelle H, Adams RP. Practical bayesian optimization of machine learning algorithms. Paper presented at: Advances in neural information processing systems 2012.

[15] Lundberg SM, Lee S-I. Consistent feature attribution for tree ensembles. arXiv preprint arXiv:170606060. 2017.

[16] Bischl B, Richter J, Bossek J, Horn D, Thomas J, Lang M. mlrMBO: a modular framework for model-based optimization of expensive black-box functions. arXiv preprint arXiv:170303373. 2017.

