# Supplementary material for: Predicting Amyloid Burden to Accelerate Recruitment of Secondary Prevention Clinical Trials
Source: J Prev Alzheimers Dis. Author manuscript; Available in PMC 2020 Dec 17. (PMC7745538; doi:10.14283/jpad.2020.44)
Supplement: 1652725_SuppMaterial [file NIHMS1652725-supplement-1652725_SuppMaterial.docx]

**Predicting amyloid burden to accelerate recruitment of secondary prevention clinical trials**

**Supplemental Material**

| **** |
| --- |
| **Supplemental Figure 1.** Predictors such as age, *APOEε*4 dose, and Clinical Function Instrument (CFI), both participant (“Pt”) and study partner (“SP”), were assumed to have monotonic relationship with amyloid PET SUVR. |

Table 1 - Model fitting algorithm steps

| **Algorithm** XGBoost – Bayesian optimization | |
| --- | --- |
| 1: | Generate an initial search space of the hyper-parameters $\boldsymbol{x}$ |
| 2: | Fit XGBoost $\boldsymbol{x}$ hyper-parameters using CV |
| 3: | Return out-of-fold predictions for each of the training data |
| 4: | Query summary metric to obtain $y$ |
| 5: | Augment data $\mathcal{D=(}\boldsymbol{x}, y)$ |
| 6: | Fit Gaussian Process Model using $D$ |
| 7: | **For** $b=1, 2, \ldots., B$ **do** |
|  | Select new $\boldsymbol{x}_{\boldsymbol{b+1}}\boldsymbol{=}{arg max}_{\boldsymbol{x}}\alpha(\boldsymbol{x}, \mathcal{D}_{b})$ |
| 9: | Fit XGBoost model $\boldsymbol{x}_{\boldsymbol{b+1}}$ hyper-parameters using CV |
| 10: | Return out-of-fold predictions for each of the training data |
| 11: | Query summary metric to obtain $y_{b+1}$ |
| 12: | Augment data $\mathcal{D}_{b+1}=(\mathcal{D}_{b}\boldsymbol{,}\left( \boldsymbol{x}_{\boldsymbol{b+1}}, y_{b+1} \right))$ |
| 13: | Update Gaussian Process Model using $\mathcal{D}_{b+1}$ |
| 14: | Fit XGBoost model using hyper-parameters such that $\boldsymbol{x}_{optim}\boldsymbol{=}{arg max}_{\boldsymbol{x}}(y)$ |
| 15: | Fit XGBoost quantile models using hyper-parameters $\boldsymbol{x}_{optim}$ |
